# Optimization of Bifunctional Antisense Oligonucleotides for Regulation of Mutually Exclusive Alternative Splicing of *PKM* Gene

**DOI:** 10.3390/molecules27175682

**Published:** 2022-09-03

**Authors:** Natalia Bartyś, Anna Pasternak, Jolanta Lisowiec-Wąchnicka

**Affiliations:** Department of Nucleic Acids Bioengineering, Institute of Bioorganic Chemistry, Polish Academy of Sciences, Noskowskiego 12/14, 61-704 Poznan, Poland

**Keywords:** alternative splicing, BASOs, *PKM* gene, hnRNP A1, bifunctional antisense oligonucleotides

## Abstract

Oligonucleotide tools, as modulators of alternative splicing, have been extensively studied, giving a rise to new therapeutic approaches. In this article, we report detailed research on the optimization of bifunctional antisense oligonucleotides (BASOs), which are targeted towards interactions with hnRNP A1 protein. We performed a binding screening assay, Kd determination, and UV melting experiments to select sequences that can be used as a high potency binding platform for hnRNP A1. Newly designed BASOs were applied to regulate the mutually exclusive alternative splicing of the *PKM* gene. Our studies demonstrate that at least three repetitions of regulatory sequence are necessary to increase expression of the PKM1 isoform. On the other hand, PKM2 expression can be inhibited by a lower number of regulatory sequences. Importantly, a novel branched type of BASOs was developed, which significantly increased the efficiency of splicing modulation. Herein, we provide new insights into BASOs design and show, for the first time, the possibility to regulate mutually exclusive alternative splicing *via* BASOs.

## 1. Introduction

The process of alternative splicing allows for the synthesis of many protein isoforms from one gene; therefore, a relatively small number of genes can produce a large number of different proteins. It is estimated that about 94% of genes with at least two exons undergo alternative splicing, increasing the diversity of proteins [1]. In addition, the formation of different isoforms is tissue-specific and is associated with various stages of organism development. The regulation of alternative splicing is a complex process and requires special sequences (cis-acting elements), to which the splicing factors (trans-acting elements) bind. These sequences may enhance or silence splicing and can be located in exons or introns. Due to these features, sequences regulating alternative splicing can be divided into exonic splicing enhancers (ESE), intronic splicing enhancers (ISE), exonic splicing silencers (ESS), and intronic splicing silencers (ISS). Moreover, the pre-mRNA secondary structure has also significant influence on alternative splicing regulation [2]. Due to a complicated regulation of the alternative splicing process, even mutations in the non-coding regions may radically change the pattern of alternative splicing. The latest literature reports show increasing number of diseases related to alternative splicing disorders [3,4,5]. New experimental methods have allowed for a broad analysis of the human genome. The results of these studies show large changes in the alternative splicing that occur during tumorigenesis [6]. The alterations in the alternative splicing induce changes in metabolism, apoptosis, control of the cell cycle, invasion, metastasis, and angiogenesis of tumor cells.

In the oligonucleotide-based technology of alternative splicing regulation, we can distinguish approaches grounded on splice-switching oligonucleotides (SSOs) and bifunctional antisense oligonucleotides (BASOs). SSOs are complementary to the sequences directly involved in the regulation of alternative splicing. Binding of SSOs to these sequences, through the Watson–Crick base pairing, prevents interactions of splicing factors and spliceosome proteins with their target sites, leading to changes in the alternative splicing pattern. Drugs based on this technology are already commercially available [7]. In contrast, BASOs have a two-part structure, i.e., they consist of an antisense part that binds to a specific site in the pre-mRNA and a regulatory part with which the selected splicing factors interact. An undeniable advantage of these molecules is that the designing process does not require detailed information about the localization of regulatory sequences which are pivotal for alternative splicing regulation of the target gene.

The idea of splicing regulation by bifunctional oligonucleotides was independently conceived in 2003 by two different research groups [8,9]. L.A. Skordis and colleagues designed BASO molecules to regulate splicing of exon 7 of the *SMN2* gene, and the regulatory part possessed a splicing enhancer sequence. In the first published research paper, the regulatory part of BASO contained repeats of 5′ GGA 3′, to which the SRSF1 factor binds [8]. Tested molecules were able to change the alternative splicing of exon 7 of the *SMN2* gene *in vitro* and in the fibroblast cell lines isolated from patients with SMA type 1. In addition to the 5′ GGA 3′ repeats, in these studies, 5′ CCGCGGA 3′, 5′ CAGACG 3′, and 5′ CACACGA 3′ SRSF1 binding sequences were also utilized [10,11]. The *SMN2* gene became the most common model for assessment of BASOs capability to modulate alternative splicing. Most of these studies were focused on using BASOs with a splicing enhancement function. The exceptions are the studies of Dickson et al., who used BASO with the regulatory sequence for hnRNP A1 binding in the regulation of *SMN2* gene splicing [12]. The hnRNP A1 protein, due to its ability to interact with both specific RNA sequences and with other proteins, is an important cellular factor. It plays a significant role in constitutive and alternative splicing, translation, mRNA trafficking, microRNA biogenesis, and telomerase maintenance [13]. hnRNP A1 is one of the most abundant nuclear proteins in the nucleus. Structurally, this protein consists of two parts. The N-terminal domain includes two tandem RRM domains, whereas the second part is a flexible glycine-rich domain [14]. The role of this protein in the modulation of alternative splicing has been well-documented. The general principle of the splicing regulation by this protein relies on binding to splicing silencer elements, located in exons or introns, and preventing binding of spliceosome proteins. Interestingly, hnRNP A1 causes the skipping of exons, in the case of the genes associated with carcinogenesis, such as *BRCA1*, *c-SRC*, and *CEACAM1* [13].

In order to silence splicing events, hnRNP A1 was the most often used protein as a target for the regulatory part of BASOs [9,12,15,16]. The first attempts were published in 2003 by Villmaire et al., and BASOs were designed to modulate alternative splicing of *Bcl-x* gene. The regulatory part was composed of two high-affinity binding sites of hnRNP A1. The same sequence was later used in 2013 by Brosseau et al. [16]. On the other hand, Dickson et al. proposed to use three repetitions of this sequence to regulate alternative splicing of the *SMN* gene. Nevertheless, detailed research has never been conducted to establish the optimal number of regulatory sequences that need to be used to achieve the modulating properties of BASOs. Based on already published data, it is not possible to compare the influence of a number of regulatory sequences on the regulatory properties of BASOs, since these molecules were used to regulate splicing of various target genes in different cell lines. Thus, detailed studies need to be performed to develop the knowledge about the optimal designing the regulatory part of BASOs. 

In this article, we aimed to optimize the composition of the regulatory part of BASOs, which can be used to regulate alternative splicing of *PKM* gene. The product of *PKM* gene expression is an enzyme involved in the last step of glycolysis [17]. The *PKM* gene undergoes mutually exclusive alternative splicing. As a result, two isoforms are produced, i.e., the PKM1 isoform that contains exon 9 and the PKM2 isoform that contains exon 10. Both isoforms differ in terms of their functions and activity. The PKM2 isoform has been found to be related with tumorigenesis. There are different mechanisms through which PKM2 contributes to cancer development and progression. First of all, PKM2 can function as a low activity dimer, leading to the accumulation of metabolic intermediates that are necessary for cells proliferation [18]. Moreover, PKM2 helps to accommodate to nutrient-limited conditions by increasing glucose uptake and lactate production. Additionally, PKM2 functions as a transcription cofactor. It was found that PKM2 can interact with various transcription factors and has an influence on the migration and invasion of cancer cells [19,20]. Knowledge about the correlation of PKM2 functions and cancer development is still under investigation. However, it is already known that PKM2 can be an attractive drug target. 

Our studies show, for the first time, the possibility to regulate the alternative splicing of the *PKM* gene by bifunctional antisense oligonucleotides. In order to achieve this aim, we present the comprehensive optimization of silencing the regulatory part of BASOs. In the presented manuscript, all RNA sequences with which the hnRNP A1 protein potentially interacts have been examined in detail. A broad range of sequences has been used to find the best ones that can be applied as a binding platform for hnRNP A1. We also performed binding affinity studies and UV melting experiments to preliminary select the full-length regulatory sequences that were used for BASOs’ construction. Herein, we reveal two regulatory sequences, for which we optimized the number of repeats that is the most effective in alternative splicing regulation. Importantly, the impact of novel BASOs on the endogenous level of the PKM isoforms in the HeLa cell line has been assessed.

## 2. Results and Discussion

### 2.1. Oligonucleotides Screening and Binding Affinity Determination

The hnRNP A1 protein contains two RNA recognition motifs (RRMs), which are responsible for interactions with RNA. Different RNA sequences were proposed as specific for each individual RRM and full-length hnRNP A1 [21,22,23]. 

**Figure 1:**
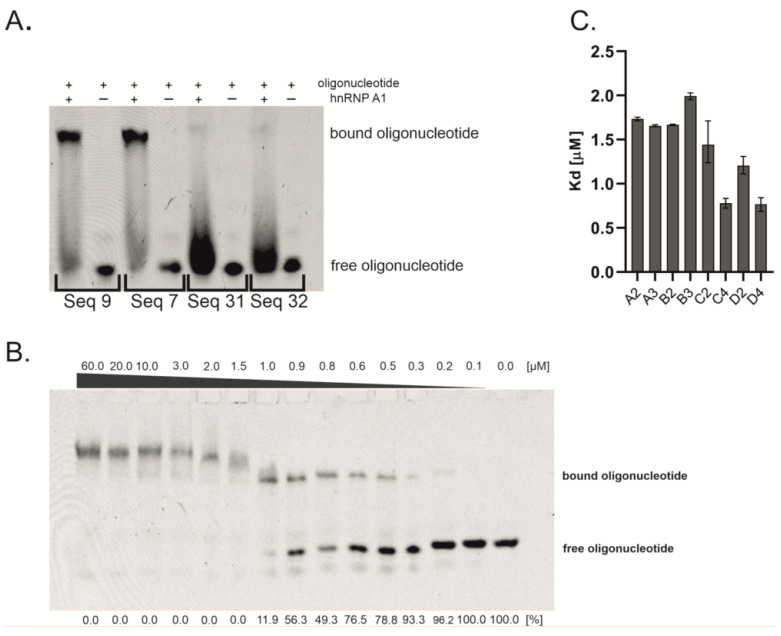
Investigations of oligonucleotides affinity to hnRNP A1. (**A**) Representative EMSA screening of oligonucleotides interacting with hnRNP A1. The oligonucleotide sequences can be found in Supplementary Material Table S1. (**B**) Representative EMSA gel for Kd determination. The amount of formed complex increases with the concentration of protein. The Kd value was calculated based on the band intensities of complex and free oligonucleotide. The number below the bottom bands presents the percentage of free oligonucleotide. The gel represents EMSA assay with C4 oligonucleotide. (**C**) The binding affinity (Kd) of oligonucleotides containing repetitions of basic sequences. The error bars present the upper and lower limits of 95% confidence intervals.

Herein, we screened 41 oligonucleotides, which were designed based on already published data, to find sequences that can be used as a binding platform of the highest potency for interactions with the hnRNP A1 protein (Supplementary Material Table S1) [22,23,24]. Almost every sequence contains the core motif (5 ′AG 3′), which seems to be necessary for interaction with hnRNP A1 [21,25]. The flanking positions can be occupied by different types of nucleotides. We aimed to assess a wide range of oligonucleotides by introducing all possible nucleotide residues in these variable positions. The screening was performed using an electrophoretic mobility shift assay (EMSA). The protein was used in high excess relative to the oligonucleotide concentration (Figure 1A). Polyacrylamide gel analysis revealed 19 sequences that interact with hnRNP A1 (Supplementary Material, Table S1). The oligonucleotides were FAM-labeled, which allowed for visualization of the formed complex and the assessment of the oligonucleotide amount involved in complex formation. Based on fluorescence intensity, we chose four oligonucleotides that were bound by the protein to the greatest extent: 5′ CAGGUAAGU 3′ (sequence A), 5′ CAGGUGAGU 3′ (sequence B), 5′ UAGGA 3′ (sequence C), and 5′ UAGGU 3′ (sequence D). Importantly, all of these sequences contain the common 5′ AGG 3′ motif, which was suggested by Beusch et al. as an optimal recognition motif for the RRM1 of hnRNP A1 (5′U_/C_AGG 3′) [21]. Moreover, sequences of oligonucleotides A and B simulate a consensus 5′ splice site. The screening confirmed the already published data, indicating that hnRNP A1 is able to bind the 5′ss sequence [22,26]. Even though all of the selected oligonucleotides formed complexes with hnRNP A1 protein, we decided to perform binding affinity determination to verify if hnRNP A1 has a stronger affinity to some of them. The dissociation constant (Kd) values could help to analyze the dependence between binding affinity and regulatory sequence efficiency in splicing regulation. Usually, a few repetitions of regulatory sequence are used for the protein-recruiting function of bifunctional antisense oligonucleotides. To date, a maximum of three repetitions of the hnRNP A1 binding motif were used to regulate alternative splicing [12]. However, it should be emphasized that the drawback of longer oligonucleotides is their ability to form secondary structures, which can have an influence on binding with the target protein. Therefore, we synthesized oligonucleotides with different repetitions of regulatory sequences and determined their binding affinity to hnRNP A1 (Table 1). The 9-nt long sequences (A, B) were repeated two (A2, B2) and three times (A3, B3), whereas the 5-nt long sequences (C, D) were repeated two (C2, D2) and four times (C4, D4). 

Kd value determination was performed by EMSA, with 14 dilutions of hnRNP A1 protein and constant oligonucleotide concentration (Figure 1B). The intensity of the formed complexes and free oligonucleotides was assessed to derive the Kd values. The results obtained from the EMSA experiments are presented in Figure 1C. The length of oligonucleotides in group A does not have an influence on binding affinity, since two-times and three-times repeated sequences possess a comparable Kd value, which is around 1.70 µM. In contrast, the Kd value in group B slightly increases with the number of sequence repetitions, from 1.76 µM for two repetitions to 1.99 µM for three repetitions. Oligonucleotides from group C and D possess the strongest affinity to protein, which increases with the number of repetitions of basic sequence. Kd values change from 1.44 µM to 0.76 µM and from 1.20 µM to 0.77 µM for groups C and D, respectively. There is a slight difference in the Kd value between oligonucleotides with two repetitions of basic sequences (C2 with sequence 5′ UAGGA 3′ vs. D2 with sequence 5′ UAGGU 3′). On the other hand, oligonucleotides with four repetitions of these sequences possess the same binding affinity (C4 and D4). To assess if the interaction of oligonucleotides with hnRNP A1 is only sequence-dependent or is also structure-dependent, we performed UV melting experiments at 260 nm and 295 nm wavelengths, which allowed us to monitor the formation of Watson–Crick and Hoogsteen H-bond-based structures, respectively. UV melting experiments showed that five out of eight oligonucleotides are able to form nucleic acids structures. Significantly, two types of structures were observed, i.e., duplex/hairpin (sequences B2 and B3) (Supplementary Material Figure S1A) and G-quadruplex (sequences D2, C4, and D4) (Supplementary Material Figure S1B), providing evidence that hnRNP A1 interacts with G-quadruplexes as well as with single-stranded and double-stranded oligonucleotides. Despite the fact that the difference in Kd values is rather minor, the observed correlation between thermodynamic studies and Kd values might suggest that the presence of G-quadruplex increases interactions with protein, in reference to single-stranded oligonucleotides (ssC2 vs. G4-forming D2). Interestingly, the structural features of G-quadruplexes might also influence the interactions with hnRNP A1. The sequences of oligonucleotides C4 and D4, which bind to the protein with the same binding affinity (Kd = 0.76 and 0.77 µM, respectively), most probably prompt the formation of intramolecular G-quadruplex structures. On the contrary, the sequence of D2 allows only for the formation of intermolecular G-quadruplex (Kd = 1.20 µM). Based on the D2, C4, and D4 sequences, all three oligonucleotides form G-quadruplexes containing similar core with two G-tetrads; however, the different molecularity of folding might induce the presence of various 3nt loop types. Thus, the preferential binding of hnRNP A1 to C4 and D4 might be due to the intramolecular character of the G-quadruplex structure and/or different loop type. Our studies stay in accordance with the observations published previously by Liu et al., who reported structure-dependent and sequence-independent interactions between hnRNP A1 and telomere G-quadruplexes and the privileged binding of intramolecular structures [27]. In contrast to groups B, C, and D, UV melting experiments do not show any transition for oligonucleotides from group A. On the other hand, both oligonucleotides from group B are able to form double-stranded structures. All the above results suggest that G-quadruplex structures might be more favorable for interaction with hnRNP A1 than single-stranded and double-stranded RNAs, however, due to minor differences in observed Kd values, further studies are required to investigate this issue in detail. 

The strongest interaction with protein was observed for oligonucleotides C4 and D4. Oligonucleotides from group C contain the 5′ GGA 3′ sequence, which is a well-known splicing enhancer motif [28,29]. Thus, we decided to exclude this group from our cell line experiments, to avoid an undesirable regulation side effect. Therefore, oligonucleotides from group D were chosen to be used as a binding platform in bifunctional antisense oligonucleotides. Additionally, oligonucleotides with one and three repetitions of sequence D were also used in the designing of BASOs, to analyze the regulation-effect dependence on the number of repeated sequences. Moreover, as a contradiction to highly structured group D, we decided also to assess the influence of single-stranded oligonucleotides on the regulatory properties of BASOs. The 5′ CAGGUAAGU 3′ sequence (oligonucleotide A) corresponds to the mammalian 5′ splice site (YAGGURAGU, where Y is pyrimidine and R is purine). In 1994, it was proposed that the 5′ splice site sequence could also be a binding site for the hnRNP A1 protein [22]. However, our studies showed that oligonucleotides that are composed of this sequence bind with weaker affinity to hnRNP A1.

### 2.2. Cell Line Results

**Figure 2:**
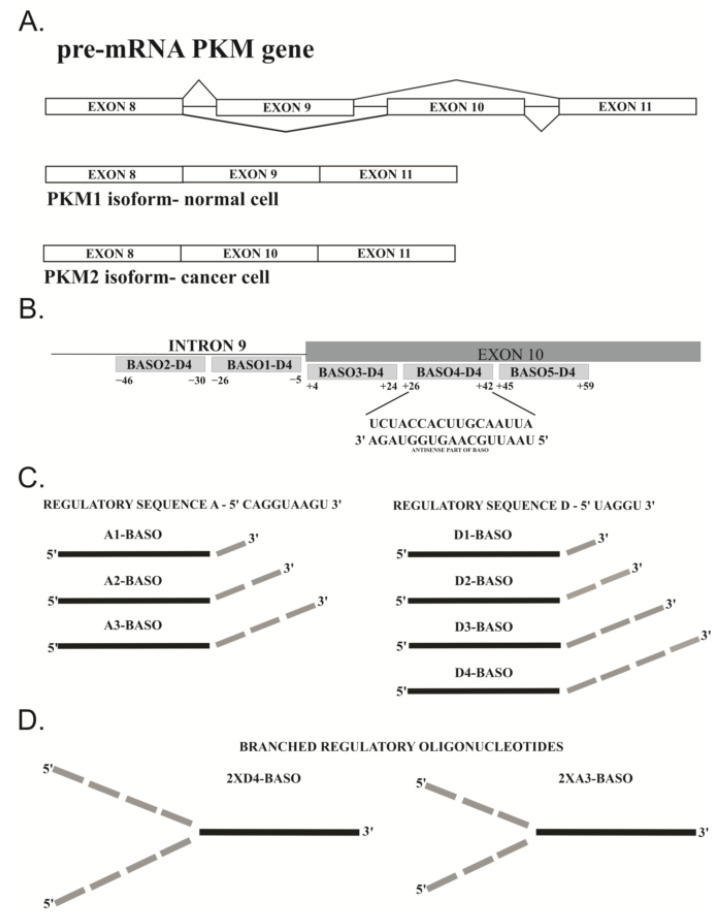
Schematic presentation of (**A**) mutually exclusive alternative splicing of *PKM* gene; and (**B**) PKM pre-mRNA fragment with different positions of BASOs hybridization *via* their antisense parts. The underlined letters represent the sequence of pre-mRNA site where the most effective BASO hybridizes. (**C**) Designed BASOs. Black rectangle represents antisense part of constant sequence that hybridizes at position +26 to position +42 of exon 10. The various regulatory parts (A1–A3 and D1–D4) are located at the 3′ end of antisense sequence (grey rectangles) and are not involved in the interactions with pre-mRNA. (**D**) The structure of branched BASO. Two regulatory sequences are linked with 5′ end of the antisense sequence.

The model for the regulation of alternative splicing by BASOs was the *PKM* gene, which contains two mutually exclusive exons, i.e., exon 9 and exon 10 (Figure 2A). In this research, we used the HeLa cell line, in which the switched expression from PKM1 to PKM2 is documented [30]. Quantitative analysis using qPCR confirmed that the level of PKM2 in HeLa cells is around 96% and the level of the PKM1 isoform is 4% (data not shown). Hitherto, research on the regulation of the alternative splicing of the *PKM* gene was focused on the splice-switching oligonucleotides (SSOs) that block regulatory sequences, to prevent their interactions with splicing factors [31]. In contrast, our attempts were focused on the BASO-mediated suppression of exon 10 splicing. The introduction of BASO with a silencing sequence should result in arrested or reduced recognition of splicing site at exon 10; thereby, exon 10 should be removed from the transcript. We designed a series of BASOs, containing oligonucleotide D4 as a regulatory part, which hybridize to various fragments of intron 9 and exon 10 to silence the splicing of exon 10 (Supplementary Material, Table S2). The transfection was made with lipofectamine as a transfection reagent. The cells were treated with 125 nM and 250 nM BASOs for 48 h. The amount of PKM1 and PKM2 isoforms was determined by qPCR. As a control, we used antisense oligonucleotides (ASOs) that hybridize to the same pre-mRNA fragments as BASOs. Additionally, the results were compared with non-transfected HeLa cell line. The initial stage of BASO optimization involved screening of the hybridization positions within the exon 10 and intron 9 of the *PKM* gene. Earlier studies on the regulation of alternative splicing of the *PKM* gene have shown that the 3′ss is essential for exon 10 definition [32]. Therefore, the studies were focused on silencing the 3′ss. Two oligonucleotides were designed to be complementary to the intron 9 in proximity to the 5′ end of exon 10 (Figure 2B). The next three oligonucleotides hybridize to the sequence in exon 10 (Figure 2B). For each BASO, the antisense part (ASO1, ASO2, ASO3, ASO4, and ASO5) was synthesized separately, to confirm whether a regulatory effect is observed due to oligonucleotide hybridization or due to the activity of the splicing factors recruited by the regulatory sequence. The effectiveness of BASOs was assessed by calculating the percentage PKM2/PKM1 ratio. The BASO that was the most effective (D4-BASO4) hybridized at position +26 to position +42 of exon 10 (see Supplementary Material Table S2 and Figure S2 for BASO sequences and quantitative results), and, for this molecule, we optimized the regulatory sequences (Figure 2C).

In already published studies, the most commonly used chemistry of antisense sequence was 2′-O-methyl-RNA (2′-O-Me-RNA) [10,16,33]. However, the regulatory sequence was used in an RNA, phosphorothioate-containing RNA (PS-RNA), DNA, or 2′-O-Me-RNA series [9,11,12,15,16]. In our research, we used the mixed chemical composition of the BASO molecule. The antisense part of each oligonucleotide was composed of the 2′-O-Me-RNA residues, whereas the regulatory part with a different length and different sequences was composed of RNA residues. Moreover, the regulatory sequence was located at the 3′ end of the BASO molecule. Our main aim was to assess the influence of a number of regulatory sequences on the modulating properties of BASOs. For this purpose, four oligonucleotides were synthesized with sequence D as a basis of the regulatory part: once repeated (D1-BASO), twice repeated (D2-BASO), three-times repeated (D3-BASO) and four-times repeated (D4-BASO) (Figure 2C).

#### 2.2.1. 5′ UAGGU 3′ Regulatory Sequence

In 1994, Burd and Dreyfuss determined, in SELEX experiments, the optimal hnRNP A1 protein binding sequence as 5′ UAGGGA/U 3′ [22]. Based on this knowledge, in the BASO experiments where the hnRNP A1 protein was treated as a target effector protein, only these two sequences (5′ UAGGGA 3′ and 5′ UAGGGU 3′) were used as a recruiting platform [12,15]. In our studies, 5′ UAGGU 3′ RNA has been shown to form a stable complex with hnRNP A1. Beutsch et al. solved the NMR structure for 5′ UUAGGUC 3′ RNA complexed with RRM1 of hnRNP A1. They showed that five nucleotides (U1 to G5) from this sequence interact with the RRM1 domain [21]. These five nucleotides almost completely overlap with oligonucleotide D (5′ UAGGU 3′), which supports our idea to use this sequence in the regulatory part of BASO. To assess the effectiveness of BASOs, we defined two parameters indicating the change of the alternative splicing of the *PKM* gene. The ratio of the PKM2/PKM1 isoforms was calculated based on the percentage level of isoforms in the pool of both isoforms. Additionally, the normalized expression of PKM1 and PKM2 was analyzed.

**Figure 3:**
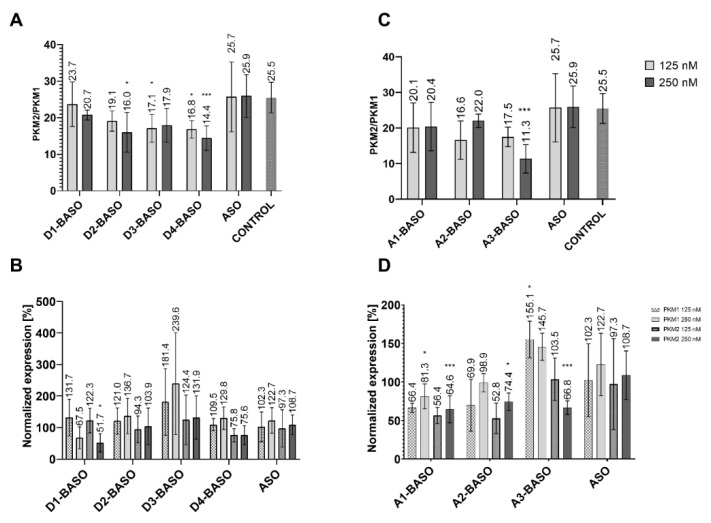
(**A**) The ratio of PKM2/PKM1 isoforms after transfection of UAGGU-based BASOs. (**B**) The PKM1 and PKM2 isoforms normalized expression after transfection of UAGGU-based BASOs. The normalized expression was calculated as a percentage of expression in non-transfected cells. (**C**) The ratio of PKM2/PKM1 isoforms after transfection of CAGGUAAGU-based BASOs. (**D**) The PKM1 and PKM2 isoforms normalized expression after transfection of CAGGUAAGU-based BASOs. The normalized expression was calculated as a percentage of expression in non-transfected cells. To obtain statistical significance, the BASOs results were compared with ASO-transfected cells. * indicates statistical significance at a level of *p* < 0.05; *** indicates statistical significance at a level of *p* < 0.01.

Each BASO was transfected in 125 nM and 250 nM concentrations. The ratio of PKM2/PKM1 isoforms in non-transfected HeLa cells was 25.5. The introduction of a BASO molecule containing one repeat of the 5′ UAGGU 3′ regulatory sequence (D1-BASO) reduced this parameter, by 1.8 for a concentration of 125 nM and by 4.8 for a concentration of 250 nM. The presence of two repeats of the 5′ UAGGU 3′ sequence within BASO (D2-BASO), at a concentration of 125 nM, changed the PKM2/PKM1 isoform ratio to 19.1. The more significant decrease in the PKM2/PKM1 ratio to 16.0 can be observed at a concentration of 250 nM. For a BASO molecule with three repeats of the regulatory sequence (D3-BASO) and four repeats (D4-BASO), at a concentration of 125 nM, the value of the PKM2/PKM1 ratio is very similar, i.e., 17.1 and 16.8, respectively. However, at a higher concentration (250 nM), a difference between these molecules can be observed, indicating that D3-BASO is less active and changes the isoform ratio by 7.6, whereas D4-BASO decreases the PKM2/PKM1 isoform ratio by 11.1 (Figure 3A). 

The results of the normalized expression of the PKM1 and PKM2 isoforms show a similar trend of changes in the alternative splicing of the *PKM* gene (Figure 3B). The shortest oligonucleotide (D1-BASO) in 125 nM concentration does not show any significant effect on expression level of both PKM isoforms. However, a two times higher concentration of D1-BASO decreases the expression of both isoforms. Importantly, the transfection of this oligonucleotide influences the expression level of both PKM isoforms, which explains the decrease in the PKM2/PKM1 ratio. A reduced PKM2/PKM1 ratio could suggest the shift of alternative splicing for higher production of PKM1 and a decrease in PKM2. However, the results of the normalized expression indicate that the change in the ratio is caused by the decrease in both isoforms, in particular by the more significant reduction in PKM2 level than in PKM1 expression. Interestingly, the level of PKM1 starts to increase proportionally to the length of the regulatory sequence. D2-BASO increased the PKM1 level by 21.0–36.7%, in reference to the isoform level within non-treated cells. The effect was similar for both BASO concentrations, suggesting that the maximum effective concentration of BASO was reached. The most effective D3-BASO is composed of three regulatory sequences. The 125 nM oligonucleotide concentration elevated PKM1 level by about 81.4%, whereas the 250 nM D3-BASO concentration caused a significant increase in PKM1 expression, which is about 240% in reference to the non-transfected HeLa cells. Interestingly, four repetitions of the 5′ UAGGU 3′ sequence within D4-BASO at both concentrations have strong influence on PKM2 expression inhibition, with the simultaneous retention of the PKM1 isoform level. Depending on what is expected, it can be assumed that the most effective BASOs are these with three or four repetitions of regulatory sequence. D3-BASO increased the PKM1 level the most significantly, however, with no influence on PKM2 expression. On the other hand, D4-BASO decreased the expression of PKM2 the most markedly, with no significant elevation of PKM1 isoform level. 

#### 2.2.2. 5′ CAGGUAAGU 3′ Regulatory Sequence

Due to the 9-nucleotide length of the 5′ CAGGUAAGU 3′ sequence, we repeated it maximum three times. The analysis of the PKM2/PKM1 isoform ratio revealed that, in each case, the introduction of BASO causes a change of this parameter (Figure 3C). A1-BASO is the least effective molecule in a series of A-BASOs in both concentrations. For the 125 nM concentration, the PKM2/PKM1 decreased to 20.1 and for 250 nM it decreased to 20.4. An additional regulatory sequence seems to enhance effectiveness of BASO and reduce the PKM2/PKM1 value to 16.6 only for 125 nM, whereas the isoform ratio was higher by about 1.6 for the 250 nM concentration, in comparison to A1-BASO. The highest potency in the modulation of the PKM2/PKM1 ratio is observed for three repetitions of the 5′ CAGGUAAGU 3′ sequence at higher oligonucleotide concentration. Although the 125 nM A3-BASO decreased the PKM2/PKM1 level to 17.5, the increased concentration resulted in the most significant change of the ratio, which was 11.3.

The results of the normalized expression of the PKM isoforms indicate that the 5′ CAGGUAAGU 3′ sequence is less effective than the 5′ UAGGU 3′ sequence. One and two repetitions of regulatory sequence are not able to increase the expression level of the PKM1 isoform, which is even lower than in non-transfected cells. On the contrary, the PKM2 isoform level is reduced more significantly than that of PKM1. The most considerable results were obtained for the three repetitions of 5′ CAGGUAAGU 3′. The A3-BASO at a 125 nM concentration increased the PKM1 level by 55%, in reference to non-transfected cells. A similar change of PKM1 expression was achieved for a 250 nM oligonucleotide concentration. However, 250 nM A3-BASO also has a significant influence on the expression of PKM2. The A3-BASO at a 125 nM concentration does not show any effect on the expression level of PKM2, whereas the transfection with a 250 nM oligonucleotide reduced the isoform level by about 33% (Figure 3D). In general, there is a possibility that A series of oligonucleotides might be bound by U1 snRNP. Gendron and coworkers used another version of 5′ss (5′ GUUGGUAUGA 3′) and suggested that the effectiveness of this sequence is related to interactions with U1 snRNP [15]. Indeed, interaction of this regulatory part with other proteins than hnRNP A1 might be a reason for the slightly weaker regulatory properties of A-BASOs. Additionally, there is also the possibility that more than one splicing protein interacts with this sequence, depending on the number of binding motifs in the regulatory part. Nevertheless, we have provided further evidence that a sequence of 5′ss can be used in the regulatory part of BASO and can inhibit the proximate 3′ss use. 

#### 2.2.3. Branched BASOs

**Figure 4:**
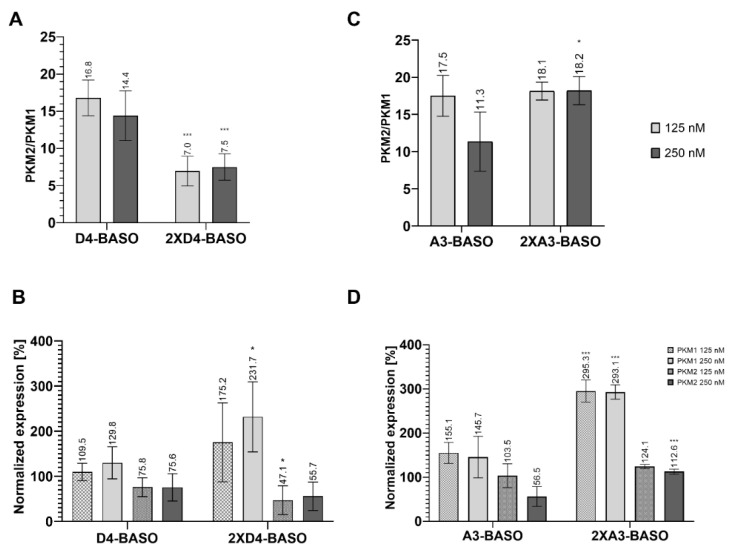
(**A**) The ratio of PKM2/PKM1 isoforms after transfection of D4-based linear and branched BASO. (**B**) The PKM1 and PKM2 isoforms normalized expression after transfection of D4-based linear and branched BASO. The normalized expression was calculated as a percentage of expression in non-transfected cells. (**C**) The ratio of PKM2/PKM1 isoforms after transfection of A3-based linear and branched BASO. (**D**) The PKM1 and PKM2 isoforms normalized expression after transfection of A3-based linear and branched BASO. The normalized expression was calculated as a percentage of expression in non-transfected cells. To obtain statistical significance, the branched BASOs results were compared with linear version of BASO. * indicates statistical significance at a level of *p* < 0.05; *** indicates statistical significance at a level of *p* < 0.01.

We also decided to design a BASO molecule that is branched with two chains of the regulatory sequence (Figure 2D). Previously, similar splicing regulatory constructs have been used *in vitro* [15]. Herein, we present the first studies of branched BASO efficiency in cell line experiments. The 2XD4-BASO includes two chains with the D4 regulatory sequence, resulting in a total of eight repeats of the 5′ UAGGU 3′ sequence within one BASO molecule. The obtained results clearly show that such branched molecules work very effectively. The ratio of the PKM2/PKM1 isoforms decreased to the greatest extent among all the tested BASOs. The presence of 2XD4-BASO reduced the isoforms ratio from 25.5 to 7.0 and 7.5 at 125 nM and 250 nM concentrations, respectively (Figure 4A). 

Notably, the significant changes in quantity of the PKM1 and PKM2 isoforms can also be noted based on their normalized expression analysis. At a 125 nM concentration of 2XD4-BASO, the level of the PKM2 isoform decreased to 47%, and the amount of PKM1 increased to 175%. The effect was even more significant at a 250 nM concentration of 2XD4-BASO, indicating a decrease in PKM2 expression to 55% and an increase in PKM1 expression to 231%, in reference to non-transfected cells. (Figure 4B). 

A similar construct was used to double the number of regulatory binding motifs of A3-BASO (Figure 2D). Surprisingly, 2XA3-BASO did not decrease the PKM2/PKM1 ratio, compared to A3-BASO. In reference to non-transfected cells, the ratio was reduced about 7.4 and 7.3 for 125 nM and 250 nM concentrations, respectively (Figure 4C). On the other hand, the normalized expression showed satisfying results. The PKM1 isoform level for both oligonucleotide concentrations was almost three times higher than in non-transfected cells. It is the best obtained result concerning the enhancement of the PKM1 level. However, in contrast to A3-BASO, none of the 2XA3-BASO concentrations reduced the PKM2 expression level. The PKM2 expression even increased to 124% and 112% for 125 nM and 250 nM concentrations, respectively (Figure 4D). The most visible difference in the effectiveness of both designed branched BASOs is their impact on the PKM2 isoform expression. 2XD4-BASO is more effective in comparison to D4-BASO, whereas 2XA3-BASO is less potent than its linear, three-times-repeated version (A3-BASO) in reducing the PKM2 level. This can support our suggestion that A series BASOs might act through the interaction with proteins other than hnRNP A1. However, it should also be considered that 2XA3-BASO contains three binding motifs, whereas there are four regulatory sequences in 2XD4-BASO. Further studies that could indicate the proteins that interact with A3-BASOs would help to understand the regulatory mechanism of these molecules.

Branched nucleic acids (bNA) have been originally designed to investigate the recognition factors of RNA branch point sequences in cells [34,35,36]. These oligonucleotides simulate the lariat, which is formed during the splicing reaction when the adenosine at the branch site triggers a nucleophilic attack on the 5′ss. This kind of molecules use both 2′ and 3′ hydroxyl groups of adenosine to form vicinal 2′-5′ and 3′-5′ phosphodiester bonds with another nucleotides. The branched oligonucleotides were proven to have splicing inhibitory properties, despite not interacting directly with pre-mRNA [34]. This strong similarity to the naturally occurring lariat causes that bNA probably recruits and sequesters branch recognition splicing factors, leading to the inhibition of the splicing reaction. Gendron et al. used such an approach to regulate alternative splicing *via* branched bifunctional antisense oligonucleotides [15]. Authors proved that using bNA with two regulatory sequences, which are not effective in linear BASOs, provides splicing silencing function. This was strong evidence that the presence of oligonucleotides with a branched adenosine can be a target for splicing factors. In our studies, we designed another type of chemical architecture to obtain a branched oligonucleotide; i.e., we applied a glycerol linker that allowed us to span the antisense part with two regulatory sequences. Therefore, from the chemical point of view, these molecules are completely different and should not be recognized by branch recognition splicing factors. In consequence, the enhanced efficiency of branched BASOs is rather due to the increased number of hnRNP A1 molecules that interact with the regulatory sequences. 

## 3. Material and Methods

### 3.1. Oligonucleotide Synthesis

All oligonucleotides were synthetized on MerMade12 synthesizerBioAutomations, LGC Biosearch Technologies, Kenning Ct Plano, USA) using β-cyanoethyl phosphoramidite chemistry and commercially available nucleoside phosphoramidites (GenePharma Co., Ltd, Suzhou, China). Oligonucleotides which were used in electrophoretic mobility shift assay were additionally 5′-labeled with 6-carboxyfluorescein (6-FAM) (ChemGenes, Wilmington, MA, USA). RNA oligonucleotides were treated with 30% ammonia/ethanol solution (Avantor Performance Materials Poland S.A., Gliwice, Poland) (3:1 *v*/*v*) and incubated at 55 °C for 18 h. The oligonucleotide solutions were then separated from the solid support and evaporated. Next, oligonucleotides were incubated with triethylamine trihydrofluoride, in the presence of dimethylformamide (Avantor Performance Materials Poland S.A., Gliwice, Poland) as a solvent, at 55 °C for 2–3 h. Oligonucleotides were precipitated in butanol, followed by sephadex column desalting. Oligonucleotides were purified via 12% polyacrylamide gel electrophoresis in denaturing conditions. The composition of all oligonucleotides was confirmed by MALDI-TOF mass spectrometry. Concentrations of oligonucleotide stock solutions were determined by UV measurements at λ = 260 nm.

### 3.2. hnRNP A1 Protein Production

Recombinant hnRNP A1 protein (2-196 aa) was produced from plasmid received as a generous gift from Frédéric Allain, at the Institute of Biochemistry, ETH Zurich, Switzerland [21]. hnRNP A1 was fused to N-terminal tag with 6 histidines and TEV-protease cleavage site. Protein was overexpressed in BL21(DE3) codon-plus (RIL)competent cells (Agilent, Santa Clara, CA, USA) in LB medium. hnRNP A1 expression was induced with 0.5 mM isopropyl-β-D-thiogalactopyranoside (IPTG) at 30 °C. Cells were harvested 16 h after induction. The cell pellet was then resuspended in lysis buffer (20 mM Na_2_HPO_4_, 0.5 mM NaCl, 15 mM imidazole, pH 7.0). Lysozyme (ThermoScientific, Waltham, MA, USA), HaltTM Protease Inhibitor Single-Use Cocktail (100×) (Thermo Scientific), and DNase I (1000 U) (EURx) were added to the cell extract, mixed for 30 min at room temperature, and sonicated afterwards (total time of sonication pulses was 4 min). Cell extract was then centrifuged for 1 h at 4 °C at 7830 rpm. To remove nucleic acids contaminants, supernatant was treated with 0.2 mg RNase A (Sigma Aldrich, Saint Louis, MO, USA) and 500U of DNase I (EURx, Gdańsk, Poland) and mixed for 1 h on ice. Supernatant was then combined with Ni-NTA resin (Thermo Scientific) and mixed for 30 min on ice. The mixture was then loaded on column, and protein was eluted with 0.3 M imidazole in a buffer (20 mM Na_2_HPO_4_, 0.5 M NaCl, pH 7.0). The 260/280 ratio was determined using NanoDrop. Protein solutions with the 260/280 ratio below 0.60 were used during the experiments. The protein was then dialyzed overnight against dialysis buffer (150 mM KCl, 50 mM L-Arg, 50 mM L-Glu, 1.5 mM MgCl_2_, 0.2 mM EDTA, 0.05% BME, 20 mM Na_2_HPO_4_, pH 7.0) and concentrated to 1.7 mg/mL final concentration using Amicon Ultra-15, NMWL: 10 kDa (Merck, Darmstadt, Germany). 

### 3.3. Electrophoretic Mobility Shift Assay (EMSA)—Oligonucleotides Screening

Each of 41 6-FAM labeled oligonucleotides was used in constant concentration of 0.5 μM and incubated with 60 μM concentration of hnRNP A1 protein. The reactions were prepared in hnRNPA1 dialysis buffer. The oligonucleotide/protein mixtures were incubated for 30 min at 4 °C. After incubation, the mixtures were centrifuged for 10 min at 14,000 rpm at 4 °C and loaded on native 4.5% polyacrylamide gel (bisacrylamide/acrylamide 37.5:1). Electrophoresis was run for 2 h, at 300 V at 4 °C. The gel was then screened in Fuji FLA-500. The intensity of complex and free RNA bands was determined by MultiGauge (FujiFilm, Tokyo, Japan). 

### 3.4. Electrophoretic Mobility Shift Assay (EMSA)-Kd Determination

The 14 dilutions of protein were prepared in the 0.1 μM to 60 μM range. Then, 6-FAM-labeled oligonucleotides were used in constant concentration of 0.5 μM. The oligonucleotide/protein mixtures were prepared in 10 μL hnRNPA1 dialysis buffer (150 mM KCl, 50 mM L-Arg, 50 mM L-Glu, 1.5 mM MgCl_2_, 0.2 mM EDTA, 0.05% BME, 20 mM Na_2_HPO_4_, and pH 7.0) and incubated for 30 min at 4 °C. After incubation, the mixtures were centrifuged for 10 min at 14,000 rpm at 4 °C and loaded on native 4.5% polyacrylamide gel (bisacrylamide/acrylamide 37.5:1). Electrophoresis was run for 2 h at 300 V at 4 °C. The gel was then screened by Fuji FLA-500 (FUJIFILM, Tokyo, Japan). The intensity of complex and free RNA bands was determined by MultiGauge. The Kd value was calculated in GraphPad Prism 8.0, and the specific binding with Hill slope function was used.

### 3.5. UV Melting Experiments

UV melting experiments were performed using the Jasco V-650 spectrophotometer (Cremella (LC) Italy) at 260 nm and 295 nm wavelengths. The oligonucleotides were dissolved in 100 mM KCl, 20 mM sodium cacodylate, and 0.5 mM Na_2_EDTA buffer (pH = 7.0) to achieve 10^−4^–10^−5^ solutions concentration. The measurements were performed using 1 mm quartz microcuvettes (30 µL) in the 85–4 °C temperature range with a 0.5 °C/min rate. The melting curves were analyzed using MeltWin 3.5 software (Copyright 1995, 1996, Jeffrey A. McDowell, New York, NY, USA).

### 3.6. Cell Culture and Oligonucleotides Transfection

HeLa cell line was cultured in RPMI 1640 medium (Gibco, Waltham, MA, USA), supplemented with vitamins, antibiotics, and 10% FBS (Gibco). Cells were seeded in 24-well plates at a density of 1 × 10^5^ cells/well, which gave 95% confluence the next day. The cells were incubated at 37 °C with 5% CO_2_ and 95% humidity. After 24 h, the medium was exchanged to antibiotic-free medium. Oligonucleotides were dissolved in OPTI-MEM (Gibco) medium with Lipofectamine 3000 (Invitrogen, Waltham, MA, USA) as a transfection reagent (1 μL of Lipofectamine and 1 µL of P3000 reagent per one reaction) and added to cells in final concentrations of 125 nM and 250 nM. Cells were harvested 48 h after transfection. All transfections were performed at least in biological triplicate. The results for each BASO were averaged.

### 3.7. RT-qPCR Analysis

The RNA from the cultured cells was isolated using acid guanidinium thiocyanate-phenol-chloroform extraction and the RNA was treated with DNase I. The quality of the isolated RNA was verified by evaluation A260/A280 and A260/A230 factors. An earlier prepared.

200 ng RNA template was used for cDNA synthesis, using the LunaScript RT SuperMix Kit (NEB). qPCR was performed on a CFX96 real-time PCR system (Bio-Rad) using Luna Universal qPCR Master Mix (NEB, Ipswich, MA, USA) and 96-well clear plates. Two pairs of target gene primers were designed to quantify the amount of PKM1 and PKM2 isoforms. Primers for PKM2 isoform amplification: 5′ ATTGCCCGTGAGGCAGAGG 3′ and 5′ TGCCAGACTTGGTGAGGACGATTA 3′. Primers for PKM1 isoform amplification: 5′ GTTCCACCGCAAGCTGTTTGAAGA 3′ and 5′ TGCCAGACTCCGTCAGAACTATCA 3′. The expression of isoforms was normalized against β-actin gene (reference gene primers: 5′ GCCAGCAGCCTCTGATCTG 3′ and 5′ CTGGTTCTTGCCAGCCTCTAG 3′). The Ct values of human β-actin gene were in the range of 17–19. The qPCR cycles were as follows: 95 °C, 1 min for predenaturation step: (95 °C, 15 s and 60 °C, 30 s) for 34 cycles.

### 3.8. qPCR Statistical Analysis

The results from replicates of particular samples were gathered to determine the mean normalized expression and its standard error of the mean (Bio-Rad CFX Manager 3.0, Hercules, CA, USA). The normalized relative expression of PKM1 and PKM2 from biological replicates for BASOs and control was compared at a significance level of 0.05 or 0.01, using Bio-rad CFX Manager 3.0 and GraphPad t-test Calculator. At least three biological and two technical repetitions were performed. The percentage level of both isoforms was used to calculate the PKM2/PKM1 ratio, which was obtained from the results of normalized expression.

## 4. Conclusions

The presented studies proved, for the first time, the possibility to regulate alternative splicing of the *PKM* gene by using bifunctional antisense oligonucleotides in the HeLa cell line. It is also the first attempt to manipulate mutually exclusive alternative splicing with these molecules. A screening assay allowed us to choose sequences that can be used in the regulatory part of BASOs. EMSA and melting experiments proved that hnRNP A1 interacts with structured oligonucleotides, and secondary structures have an impact on the binding affinity. Based on our cell line studies, it can be assumed that the effectiveness of the regulatory part of BASOs is dependent on the type of binding motif. Moreover, we noticed differences in the effectiveness of the PKM1 and PKM2 level modulation, for both used series of BASOs. Therefore, efforts must be made to carefully choose the regulatory sequence. Furthermore, the number of regulatory binding motifs has an influence on the regulatory properties of BASOs. Both studied sequences were the most effective in increasing the PKM1 level, when three repetitions were used in the regulatory part. However, the elevated PKM1 level was not always simultaneous with reduced PKM2 level. Additionally, the doubling of both regulatory parts in branched BASOs, to the greater extent, increased the production of PKM1. On the other hand, only 2XD4-BASO significantly influenced the PKM2 level. Therefore, experiments on the optimization and effectiveness of these molecules are pivotal to be able to design simple and potent therapies based on BASOs.

The analysis of the splicing regulatory potential of BASOs containing different sequences and repetitions of regulatory sequences, as presented herein, might facilitate the design and development of novel, efficient therapeutic tools. To date, a few oligonucleotide drugs based on SSO molecules have already been approved. However, the designing of SSOs requires very careful and detailed investigations on the regulatory sequences, which are pivotal for the splicing regulation of the target pre-mRNA. BASO molecules are potentially more optimal tools in regulating the splicing in all diseases and disorders related to splicing, in comparison to SSOs, due to the less stringent requirements for the site of the hybridization with pre-mRNA. In addition, the effective optimization of the structure of BASOs can allow for using the same regulatory part to regulate the alternative splicing of different genes. 

## Figures and Tables

**Table 1:** Oligonucleotides that consist of basic sequence repetitions, which were found to be preferentially recognized by hnRNP A1 protein.

Name	Sequence (5′ -> 3′)
A2	CAGGUAAGU CAGGUAAGU
A3	CAGGUAAGU CAGGUAAGU CAGGUAAGU
B2	CAGGUGAGU CAGGUGAGU
B3	CAGGUGAGU CAGGUGAGU CAGGUGAGU
C2	UAGGA UAGGA
C4	UAGGA UAGGA UAGGA UAGGA
D2	UAGGU UAGGU
D4	UAGGU UAGGU UAGGU UAGGU

## Data Availability

Not applicable.

## Supplementary Materials

The following supporting information can be downloaded at: https://www.mdpi.com/article/10.3390/molecules27175682/s1. Table S1: Screening results of binding motifs for hnRNP A1; Table S2: The list of D4-BASO sequences used to optimize position of hybridization with *PKM* gene; Figure S1: The graphs present the melting curves of regulatory oligonucleotides; Figure S2: The ratio of PKM2/PKM1 isoforms after cells treatment with BASOs and ASOs that hybridize at different positions of PKM pre-mRNA.

## Sample Availability

Samples of the compounds are not available from the authors.
