# Supplementary material for: Optimization of Bifunctional Antisense Oligonucleotides for Regulation of Mutually Exclusive Alternative Splicing of PKM Gene
Source: Molecules. 2022 Sep 3;27(17):5682. doi: 10.3390/molecules27175682 (PMC9457596; doi:10.3390/molecules27175682)
Supplement: Supplementary file 1 [file molecules-27-05682-s001.zip › molecules-1866201-supplementary.pdf]

SUPPLEMENTARY MATERIAL

Optimization of bifunctional antisense oligonucleotides for regulation of mutually exclusive alternative splicing of *PKM* gene

*Natalia Bartyś, Anna Pasternak and Jolanta Lisowiec-Wąchnicka **

Department of Nucleic Acids Bioengineering, Institute of Bioorganic Chemistry, Polish Academy of Sciences, Noskowskiego 12/14, 61-704 Poznan, Poland

*To whom correspondence should be addressed. Tel: + 48 618 528 503; Email: lisowiec@ibch.poznan.pl (J.L.W.);

Table S1. Screening results of binding motifs for hnRNP A1. The crosses indicate the amount of oligonucleotide involved in complex formation: ++++ corresponds to 100%-85% of oligonucleotide, +++ corresponds to 85%-70% of oligonucleotide, ++ corresponds to 70%-50% of oligonucleotide, + corresponds to <50% of oligonucleotide, n.b.- no binding

**Name**	**Sequence 5′→3′**	**Amount of oligonucleotide involved in complex formation**
1. (sequence A)	CAGGUAAGU	++++
2. (sequence B)	CAGGUGAGU	++++
3. (sequence C)	UAGGA	++++
4. (sequence D)	UAGGU	++++
5.	UAGG	++++
6.	AAGGUAAGU	+++
7.	UGUAGG	+++
8.	CGUAGG	++
9.	UAUAGG	++
10.	UGCAGG	++
11.	UUCAGG	++
12.	UAGA	++
13.	CACAGG	+
14.	CAUAGG	+
15.	CGCAGG	+
16.	GCUAGA	+
17.	UACAGG	+
18.	UUUAGA	+
19.	UUUAGG	+
20.	AAGGUGAU	n.b.
21.	CACAGA	n.b.

22.	CCCAGA	n.b.
23.	CCCAGG	n.b.
24.	CCUAGA	n.b.
25.	CCUAGG	n.b.
26.	CGCAGA	n.b.
27.	CUCAGA	n.b.
28.	CUCAGG	n.b.
29.	CUUAGA	n.b.
30.	CUUAGG	n.b.
31.	CAUAGA	n.b.
32.	UACAGA	n.b.
33.	UAUAGA	n.b.
34.	UCCAGA	n.b.
35.	UCCAGG	n.b.
36.	UCUAGA	n.b.
37.	UCUAGG	n.b.
38.	UGCAGA	n.b.
39.	UGUAGA	n.b.
40.	UUCAGA	n.b.
41.	AUUUA	n.b.

Table S2. The list of D4-BASO sequences used to optimize position of hybridization with *PKM* gene. Each antisense part was used with D4 sequence ($(UAGGU)_4$) as a regulatory part. The PKM2/PKM1 ratio was assessed for 250 nM BASOs concentration. * indicates the same regulation effect as observed for corresponding ASO.

**BASO sequence 5′→3′**	**Hybridization position within *PKM* gene**	**PKM2/PKM1**
GAGAGGGGGACAGAGCU-$(UAGGU)_4$	Intron 9 3'ss -5 -26	36.3
GGGAGCAACAUCCGUCCAGAGG-$(UAGGU)_4$	Intron 9 3'ss -30-46	34.0
GGCAGCCUCUGCCUCACGGG-$(UAGGU)_4$	Exon 10 3'ss +4+24	2.0*
UAAUUGCAAGUGGUAGA-$(UAGGU)_4$	Exon 10 3'ss +26+42	14.4
CGGCGGAGUUCCUCA-$(UAGGU)_4$	Exon 10 3'ss +45+59	20.1

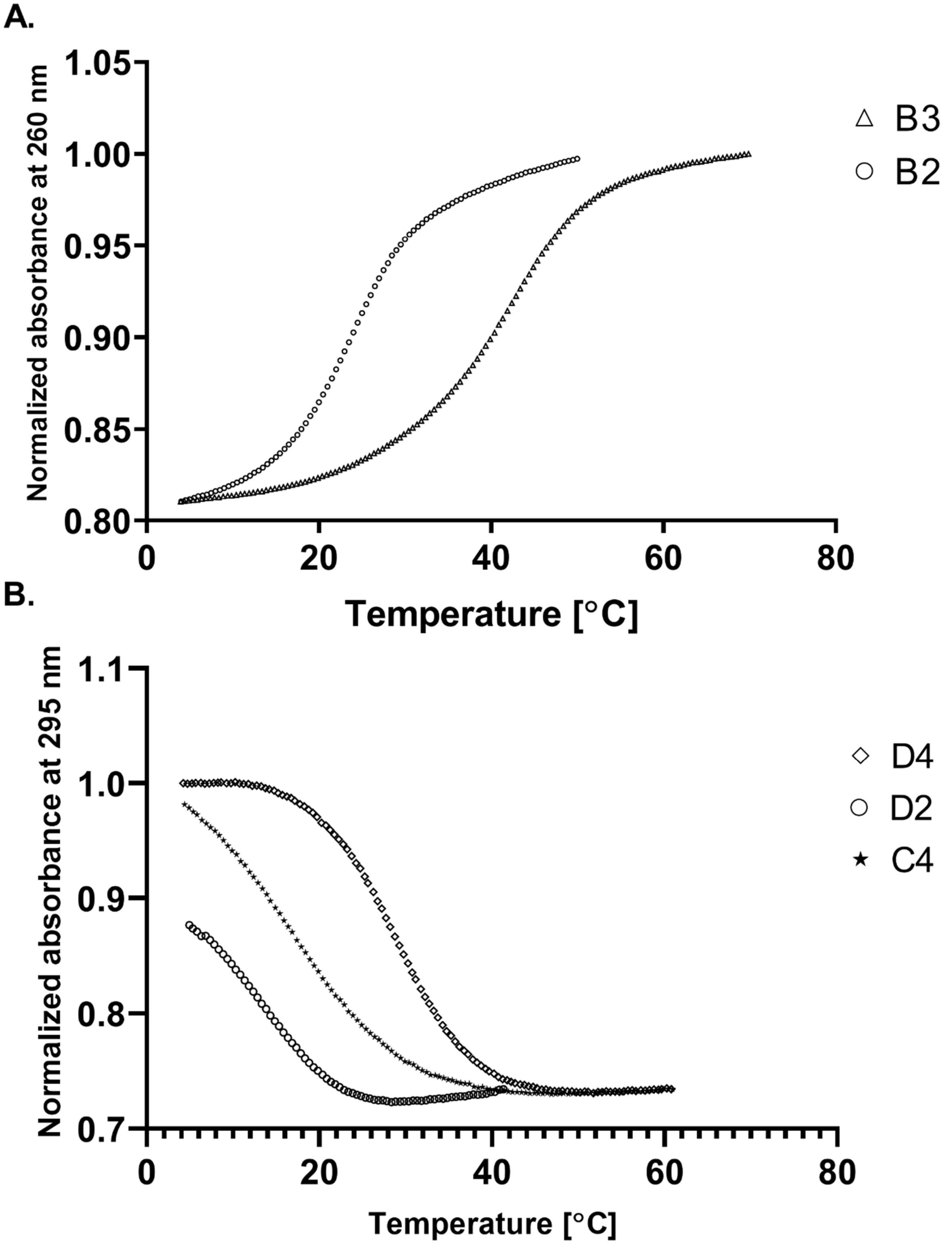

Figure S1. The graphs present the melting curves of regulatory oligonucleotides. **A.** The melting curves for double-stranded structures at 260 nm. **B.** The melting curves for G-quadruplexes at 295 nm

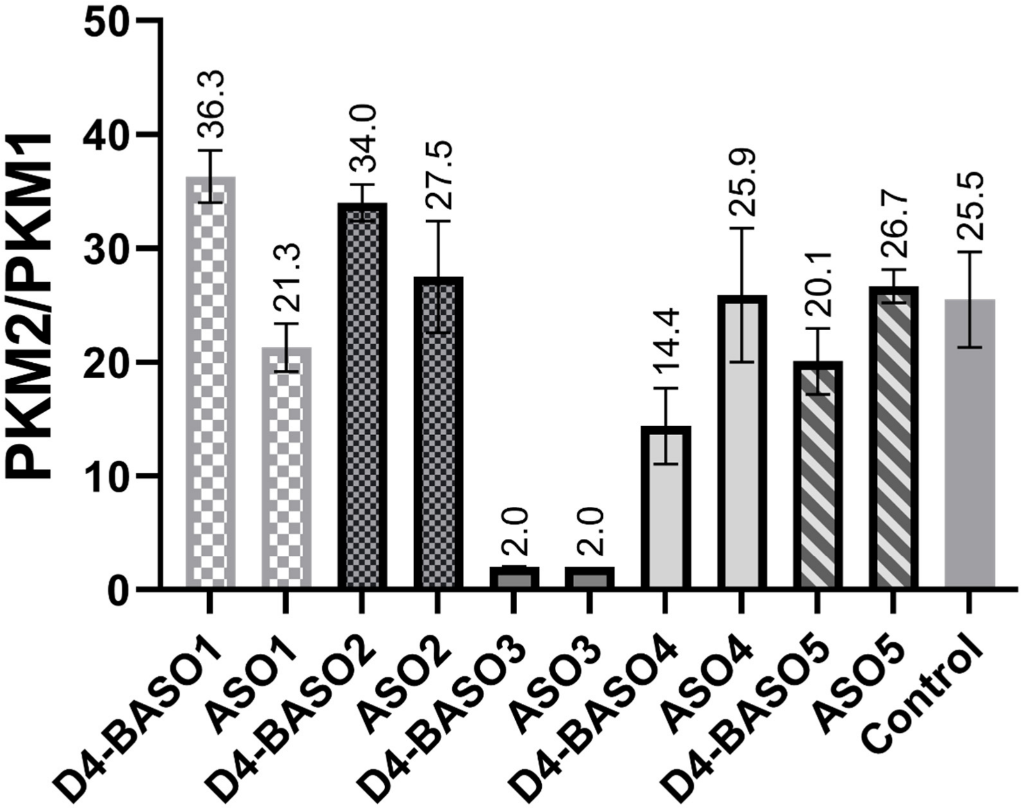

Figure S2. The ratio of PKM2/PKM1 isoforms after cells treatment with 250 nM concentration of BASOs and ASOs that hybridize at different positions of *PKM* pre-mRNA.
